# Associations between MRI features versus knee pain severity and progression: Data from the Vancouver Longitudinal Study of Early Knee Osteoarthritis

**DOI:** 10.1371/journal.pone.0176833

**Published:** 2017-05-04

**Authors:** Eric C. Sayre, Ali Guermazi, John M. Esdaile, Jacek A. Kopec, Joel Singer, Anona Thorne, Savvas Nicolaou, Jolanda Cibere

**Affiliations:** 1 Arthritis Research Canada, Richmond, BC, Canada; 2 Radiology, Boston University School of Medicine, Boston, MA, United States of America; 3 Medicine, University of British Columbia, Vancouver, BC, Canada; 4 Medicine, University of Calgary, Calgary, AB, Canada; 5 School of Medicine, University of Queensland, Brisbane St. Lucia, QLD, Australia; 6 School of Population & Public Health, University of British Columbia, Vancouver, BC, Canada; 7 Radiology, University of British Columbia, Vancouver, BC, Canada; University of Umeå, SWEDEN

## Abstract

**Objective:**

To determine associations between features of osteoarthritis (OA) on MRI and knee pain severity and knee pain progression.

**Design:**

Baseline, 3.3- and 7.5-year assessments were performed for 122 subjects with baseline knee pain (age 40–79), sample-weighted for population (with knee pain) representativeness. MRIs were scored for: osteophytes (0:absent to 3:large); cartilage (0:normal to 4:full thickness defect; 0/1 collapsed); subchondral sclerosis (0:none to 3:>50% of site), subchondral cyst (0:absent to 3:severe), bone marrow lesions (0:none to 3:≥50% of site); and meniscus (0:normal to 3:maceration/resection), in 6–8 regions each. Per feature, scores were averaged across regions. Effusion/synovitis (0:absent to 3:severe) was analyzed as ≥2 vs. <2.

Linear models predicted WOMAC knee pain severity (0–100), and binary models predicted 10+ (minimum perceptible clinical improvement [MPCI]) and 20+ (minimum clinically important difference [MCID]) increases. Models were adjusted for age, sex, BMI (and follow-up time for longitudinal models).

**Results:**

Pain severity was associated with osteophytes (7.17 per unit average; 95% CI = 3.19, 11.15) and subchondral sclerosis (11.03; 0.68, 21.39). MPCI-based pain increase was associated with osteophytes (odds ratio per unit average 3.20; 1.36, 7.55), subchondral sclerosis (5.69; 1.06, 30.44), meniscal damage (1.68; 1.08, 2.61) and effusion/synovitis ≥2 (2.25; 1.07, 4.71). MCID-based pain increase was associated with osteophytes (3.79; 1.41, 10.20) and cartilage defects (2.42; 1.24, 4.74).

**Conclusions:**

Of the features investigated, only osteophytes were consistently associated with pain cross-sectionally and longitudinally in all models. This suggests an important role of bone in early knee osteoarthritis.

## Introduction

Osteoarthritis (OA) is the most common form of arthritis among Canadians, affecting 11% of the 2001 population, and nearly one third of those aged 65–69.[1] More recently, among U.S. adults, nearly 27 million had clinical osteoarthritis in 2008 (up from 21 million in 1995).[2] Being strongly related to age and body mass index (BMI), with the increasing average age and adiposity of these populations, OA constitutes a substantial increasing public health burden.[3–6]

Kellgren-Lawrence (KL) grade, a 5-level index ranging from 0 to 4, is a standard radiographic measurement of joint deterioration used in diagnosing and staging of OA.[7] Additionally, OA can be characterized by an assortment of features detectable on MRI, including osteophytes, cartilage defects, subchondral sclerosis, subchondral cysts, bone marrow lesions (BMLs), meniscal damage and effusion. Most previous investigations of the potential association between knee pain and these features were cross-sectional, with only a few (excluding systematic reviews so as not to double count studies) reporting longitudinal results,[8–12] or population-based findings.[9, 13–18] Findings are not entirely consistent.[19] Some studies found associations between knee pain and osteophytes,[8, 9, 11, 18, 20] cartilage defects,[9, 11, 17, 18, 20–22] subchondral sclerosis,[15, 16, 18, 21–23] subchondral cysts,[15, 18] BML,[9, 11, 13, 15, 17, 18, 22–27] meniscal damage,[11, 14, 22] or effusion.[18, 22–25, 27, 28] In contrast, some investigated but did not find associations between knee pain and osteophytes,[15, 27, 28] cartilage defects,[15, 27, 29] subchondral cysts,[9, 27] BML,[10, 12, 28] meniscal damage,[15, 23, 27–29] or effusion.[29]

In light of these mixed results, here we aim to clarify the associations between both knee pain severity and progression versus the MRI features osteophytes, cartilage defects, subchondral sclerosis, subchondral cysts, bone marrow lesions, meniscal damage and effusion/synovitis (combined due to non-separability on non-contrast MRI), in a population-based cohort with knee pain with 7.5 years of follow-up. As our study reports results from both cross-sectional and longitudinal models together, as well as reporting cross-sectional results for severity of pain rather than only presence or absence of pain, we aim to build a more complete picture of the implications of MRI features on knee pain as a whole—inclusive of both severity and progression.

## Materials and methods

### Ethics approval

This study was conducted in accordance with the declaration of Helsinki and was approved by the Clinical Research Ethics Board of the University of British Columbia. All participants gave written informed consent at all three time points.

### Data collection

Source data came from a longitudinal study conducted in Vancouver, Canada,[30] a population-based cohort of individuals aged 40 to 79 with knee pain “on most days of the month at any time in the past and any pain in the past 12 months.” Data collection for the source study has been previously described.[31, 32] Subjects were excluded at baseline if they had inflammatory arthritis or fibromyalgia, previous knee arthroplasty, knee injury or surgery within the previous 6 months, knee pain referred from hips or back or were unable to undergo MRI. The examination was performed by an experienced rheumatologist (JC). We have previously reported in this cohort that, based on MRI cartilage damage and x-ray findings, 13% had no OA, 49% had pre-radiographic OA (cartilage damage but KL<2), and 38% had radiographic OA.[32] Based on power calculations for the original source study,[30] this cohort enrolled 255 individuals, stratified by age decade and sex in roughly equal group sizes (stratum sizes were capped at 35) to ensure adequate sample size across the age-sex spectrum. Baseline visits occurred between 2002 and 2005. A random list of households was obtained from the telephone directory listings, which included address and telephone information. Invitation letters were mailed to randomly selected households. This was followed by standardized telephone screening for preliminary eligibility, followed by in-person detailed eligibility screening. Once adequate numbers were attained in a given age-sex group, data collection stopped within that group. The first time this happened, the sample collected to that point provided the estimated population proportional distribution of age and sex in those with knee pain. This distribution was used to develop a sample weight (see below). Collection continued until sufficient numbers were reached in each group.[32]

**Fig 1:**
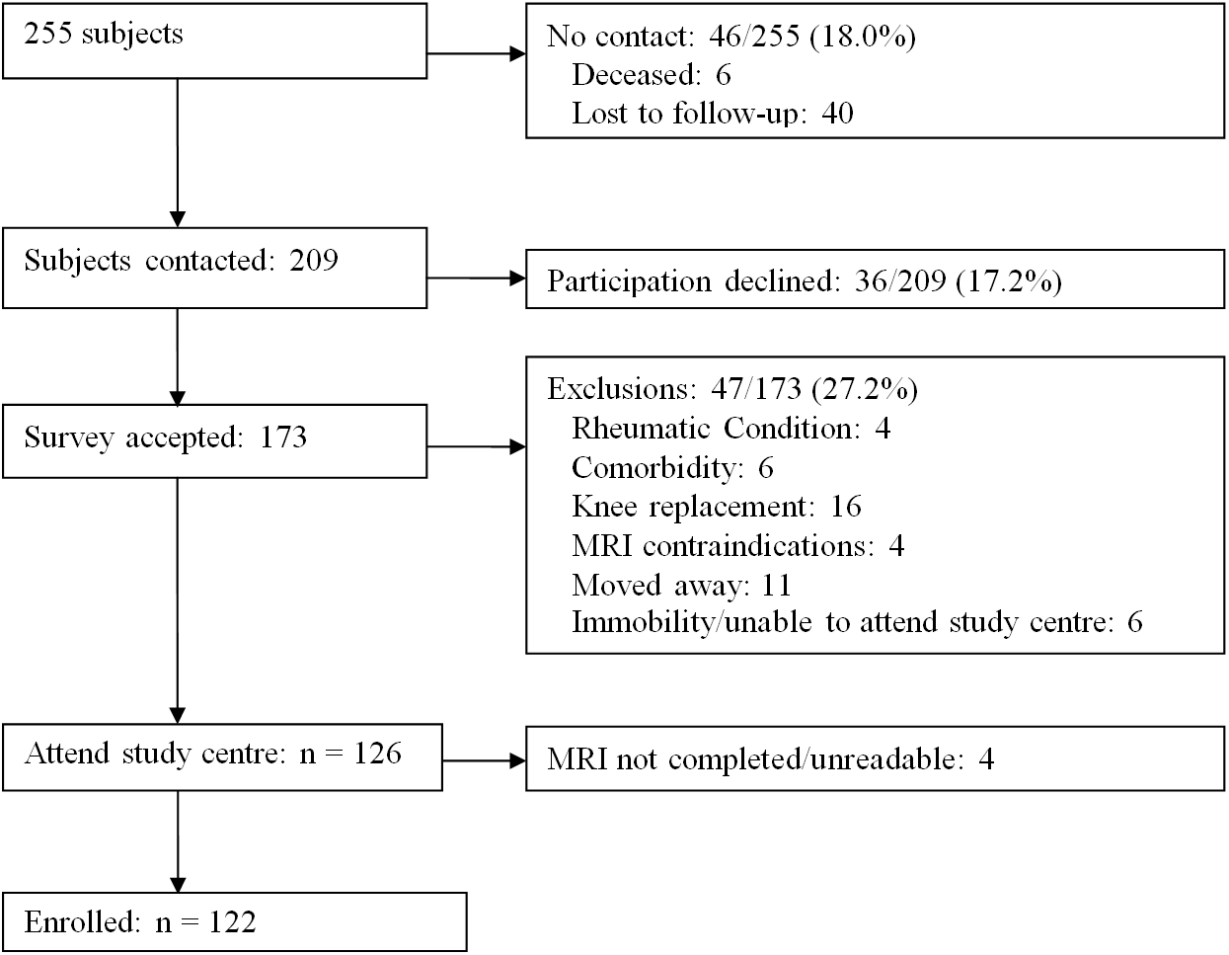
6-year follow-up (VALSEKO) recruitment flow chart.

In addition to the baseline cycle, two follow-up cycles were undertaken, at weighted mean 3.3 (SD 0.6) and 7.5 (SD 0.6) years. The present study includes the N = 122 subjects who were available at the third cycle (the second follow-up cycle), although only 108 were available to provide data in the middle cycle. The remainder of the baseline sample who were not available for the third visit dropped out for a variety of reasons (Fig 1), including deceased (n = 6), other no contact (40), declined participation (36), exclusions for rheumatic conditions (4), comorbidity (6), knee replacement (16), MRI contraindication (4), moved away (11), immobility (6), or MRI completed but not readable (4).

The study knee was the more painful knee at baseline. MRIs were acquired on a GE 1.5T magnet at a single centre using a transmitter-receiver extremity knee coil. The imaging protocol included four MRI sequences, as previously described.[30, 32] 1) Fat suppressed T1-weighted three-dimensional (3D) spoiled gradient echo (SPGR) sequence with images obtained in the sagittal plane and reformatted images in the axial and coronal planes; 2) fat suppressed T2-weighted fast spin echo (FSE) sequence with images obtained in the coronal plane; 3) T1-weighted FSE sequence with images obtained in the oblique sagittal plane (angulated according to the course of the anterior cruciate ligament); and 4) T2-weighed FSE sequence with images obtained in the oblique sagittal plane (angulated according to the course of the anterior cruciate ligament). MRIs were scored by a board-certified musculoskeletal radiologist (AG) with 15 years of semiquantitative reading experience, who was blinded to clinical, radiographic, and time sequence information. Osteophytes (0: absent, 1: small, 2: moderate, 3: large) were scored in 8 regions: lateral and medial femur, lateral and medial tibia, and lateral, medial, superior and inferior patella. Cartilage, subchondral sclerosis, subchondral cyst, and BML were scored in 6 regions: lateral and medial femur, lateral and medial tibia, patella and trochlear groove. Cartilage was graded on a 0–4 semi-quantitative scale based on the following definitions, previously described by Disler et al:[33] 0: normal, 1: abnormal signal without cartilage contour defect, 2: contour defect of < 50% cartilage thickness, 3: contour defect of 50–99% cartilage thickness, 4: 100% cartilage contour defect with subjacent bone signal abnormality. (0 and 1 were collapsed since 1 represents signal hyperintensity on T2-weighted images of indeterminate significance.) Subchondral sclerosis was scored at baseline from 0 to 3 for each region of the knee as: 0: none, 1: <25% of the subregion, 2: 25–50% of the subregion, 3: >50% of the subregion. Subchondral cyst was scored as: 0: absent, 1: mild, 2: moderate, 3: severe. BML was scored as: 0: none; 1: <25% of site; 2: 25%–49% of site; and 3: ≥ 50% of the site. Meniscal damage was scored as: 0: normal, 1: intra-substance signal, 2: tear, 3: maceration/resection. Meniscal damage was scored in the following 6 regions: lateral anterior, lateral body, lateral posterior, medial anterior, medial body and medial posterior. Intra-rater reliability analyses were previously performed on the scoring of each surface within each feature. The intraclass correlation coefficients (ICCs) were as follows: osteophytes 0.77–0.89, cartilage 0.84–1.00, subchondral sclerosis (excluding patella) 0.63–0.79, subchondral sclerosis (patellar) 0.31, subchondral cyst (excluding patella) 0.61–0.78, subchondral cyst (patellar) 0.50, BML 0.81–0.93, meniscus 0.60–0.83, and effusion/synovitis (0–3 scale) 0.76.

For each feature other than effusion/synovitis, region scores were added up and the sum divided by the number of regions to form region-average scores. Effusion/synovitis (0: absent, 1: mild, 2: moderate, 3: severe) was scored for the overall knee and analyzed as ≥2 vs. <2. Grades 0 and 1 were collapsed because MRI effusion/synovitis score 1 may reflect physiologic joint fluid. Grades 2+ were combined because there was only a single grade 3 effusion in the baseline source data, and in fact none in the longitudinal sample. Western Ontario and McMaster Universities Osteoarthritis Index (WOMAC) knee pain scale (normalized 0–100; higher numbers worse) was administered at each time point.[34]

For use in sensitivity analyses, we collected self-reported data on medications, with the following question: “Please list all current medications that you are taking below, including the dose or how much of the medication you take, and how often you take it (for example NAME: Glucosamine, DOSE: 500mg, HOW OFTEN: twice a day). Please note that the types of medications have been divided into prescribed, over the counter, herbal therapies/supplements, and vitamins and minerals.” We also collected self-reported data on physiotherapy use, with the following question: “Please mark all other or alternative therapies that you have used for your knee pain at any time in the past or currently. Indicate the time period of use and how often you have used it on average.” [with one category labeled “Physiotherapy”].

### Statistical methods

In order to obtain results that were population-representative, a sample weight was developed for the baseline sample as the ratio of population proportion in a given age-sex cell over the baseline sample proportion in that cell. The weight was scaled to sum to the baseline sample size (255). The longitudinal subset analyzed in the present study consisted of 122 subjects followed up from the original baseline sample to the third cycle.[30] A sample weight was developed for the longitudinal sample as the ratio of baseline sample proportion in a given age-sex cell over the longitudinal sample proportion in that cell, multiplied by the baseline sample weight, thus retaining the population representativeness in this subsample. The sample weight was scaled to sum to the follow-up sample size (122). All analyses in the present study were weighted with the longitudinal sample weight.

Spearman rank correlations were computed among the MRI features at baseline. Cross-sectional models predicting WOMAC pain severity were fit on three time cycles simultaneously (baseline, 3.3-year and 7.5-year follow-up; N = 366), using generalized estimating equations (GEE) to account for correlated data. Longitudinal (change) models of indicators for 10+ units increase in WOMAC pain (based on the minimum perceptible clinical improvement of 9.7 [MPCI]),[35] and 20+ units increase (based on the minimum clinically important difference of 19.9 [MCID]),[36] were fit on both 2-cycle changes simultaneously (cycles 1–2 and 2–3; N = 244), using GEE for binary regression. Separate models were fit with each MRI feature as predictor, adjusting for age, sex, BMI (and follow-up time for longitudinal models). Binary model fit was assessed via the Hosmer and Lemeshow goodness of fit (GOF) test.[37] Choice of the working correlation structure in continuous models was assessed via the quasi information criterion (QIC).

We performed several sensitivity analyses. First, we adjusted all models for self-reported current regular non-steroidal anti-inflammatory drug (NSAID) or acetaminophen use, and self-reported current or recent physical therapist (PT) use. Next, we explored the associations between the individual surface cartilage scores (dichotomized per surface into full thickness lesion or not) and our outcomes variables. We also performed two sensitivity analyses exploring possible alternatives to our aggregate osteophyte score: one eliminating medial/lateral patellar surfaces, the other eliminating superior/inferior patellar surfaces. Finally, we compared the followed up subsample of 122/255 to the subsample that was not followed up (133/255), on baseline values of the MRI features of interest, age, sex, and BMI.

Analyses were performed using SAS version 9.4 (SAS Institute Inc., Cary, NC, USA).

## Results

**Fig 2:**
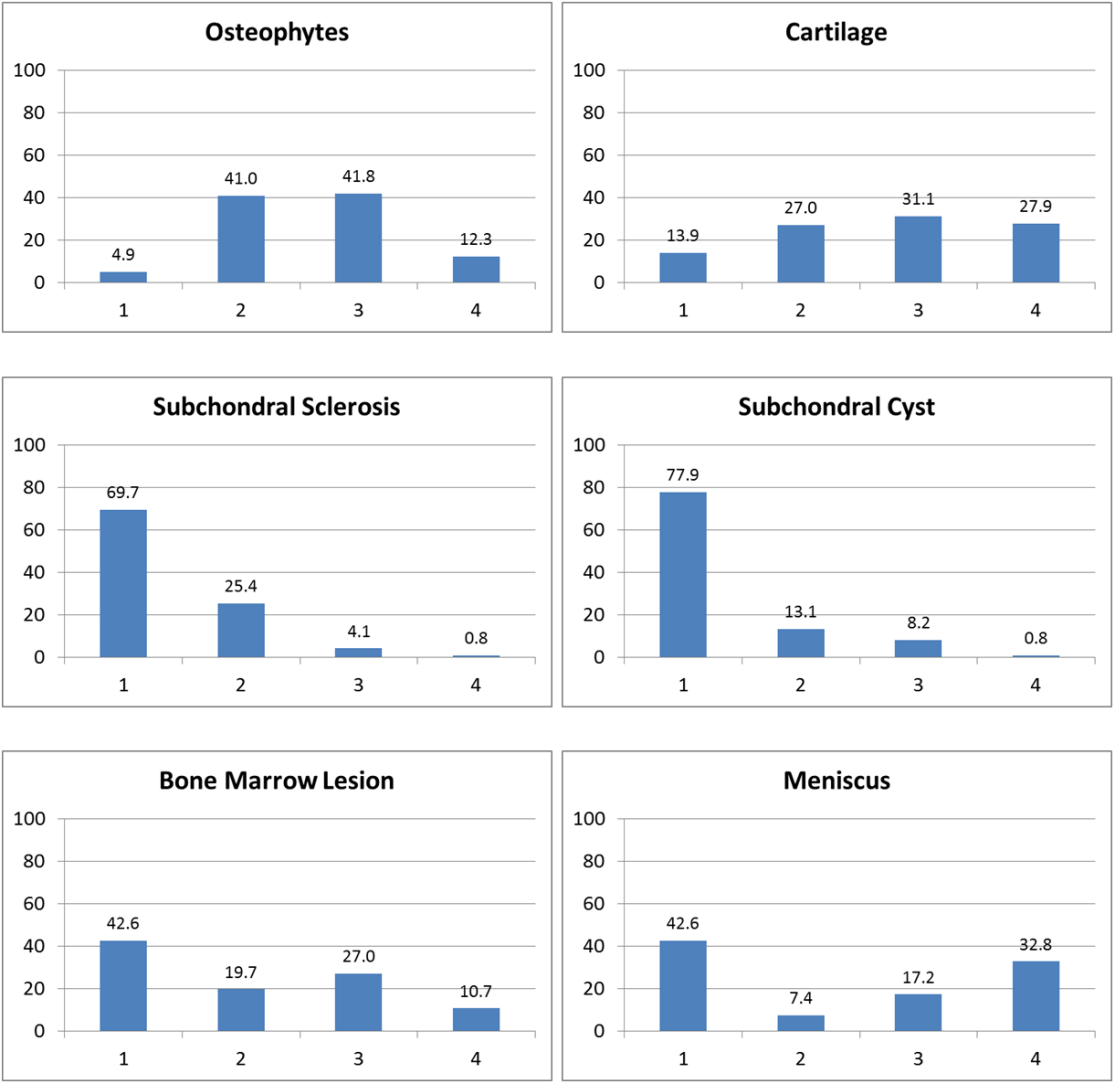
Distribution of within-subject feature score maxima at baseline (%).

Weighted characteristics of the sample are described in Table 1. 55.7% were female. At baseline, average age (SD) was 55.5 (9.1), mean BMI was 26.1 (range = 18.7–37.9; SD = 4.0), and 39.6% had radiographic OA (KL grade ≥2). Mean region-average scores at baseline were 0.93 (SD = 0.47) for osteophytes, 1.74 (0.59) for cartilage, 0.08 (0.16) for subchondral sclerosis, 0.06 (0.15) for subchondral cysts, 0.27 (0.32) for BML, and 0.48 (0.64) for meniscus. 29.6% had baseline effusion score ≥2. Mean baseline WOMAC pain was 19.1 (SD = 17.3). Spearman rank correlations among the seven MRI features were all statistically significant, ranging from 0.21 (subchondral cyst vs. meniscus) to 0.68 (osteophyte vs. cartilage). Mean region-average scores at final follow-up (cycle 3) were 1.17 (SD = 0.57) for osteophytes, 2.04 (0.76) for cartilage, 0.18 (0.31) for subchondral sclerosis, 0.13 (0.19) for subchondral cysts, 0.47 (0.48) for BML, and 0.65 (0.73) for meniscus. 45.9% had effusion /synovitis score ≥2. Mean WOMAC pain was 19.6 (SD = 20.5). Fig 2 shows the distribution of the six within-subject feature maxima at baseline.

**Table 1:** Weighted sample characteristics, pain and MRI features by cycle.

MRI feature	Baseline (Weighted N = 122.0)	1st Follow-up (Weighted N = 108.6)	2nd Follow-up (Weighted N = 122.0)
Female, n (%)	68.0 (55.7)	59.9 (55.1)	68.0 (55.7)
Age	55.5 (9.1)	59.0 (8.8)	63.0 (9.0)
BMI	26.1 (4.0)	26.1 (4.0)	26.3 (4.4)
KL grade, n (%)			
0	49.7 (40.8)	26.8 (24.6)	36.7 (30.1)
1	24.0 (19.7)	23.7 (21.8)	20.6 (16.9)
2	27.4 (22.5)	28.2 (26.0)	21.6 (17.7)
3	13.8 (11.3)	13.4 (12.3)	21.0 (17.2)
4	7.1 (5.8)	16.6 (15.3)	19.5 (16.0)
Missing	0.0 (0.0)	0.0 (0.0)	2.6 (2.1)
WOMAC pain	19.1 (17.3)	15.0 (15.9)	19.6 (20.5)
Osteophytes (sum/8)^1^	0.93 (0.47)	1.00 (0.48)	1.17 (0.57)
Cartilage (sum/6)^1^	1.74 (0.59)	1.84 (0.64)	2.04 (0.76)
Subchondral sclerosis (sum/6)^1^	0.08 (0.16)	0.12 (0.21)	0.18 (0.31)
Subchondral cysts (sum/6)^1^	0.06 (0.15)	0.08 (0.16)	0.13 (0.19)
BML (sum/6)^1^	0.27 (0.32)	0.29 (0.36)	0.47 (0.48)
Meniscus (sum/6)^1^	0.48 (0.64)	0.56 (0.71)	0.65 (0.73)
Effusion/synovitis ≥2, n (%)	36.1 (29.6)	51.3 (47.2)	56.0 (45.9)

Mean (SD) or otherwise indicated

^1^ Average grade across regions

Table 2 summarizes the progression of WOMAC pain as a continuous variable as well as via binary indicators for 10+ and 20+ units increase in pain. Average WOMAC pain remained fairly constant relative to its SD (changes between cycles 1–2, 2–3 and 1–3 respectively were -4.3, 4.5 and 0.7). However, subject-level pain could vary in either direction. Between cycles 1–2, 14.1% progressed by at least 10 units, and 6.9% progressed by at least 20 units. Between cycles 1–3, 22.3% progressed by at least 10 units, and 14.7% progressed by at least 20 units.

**Table 2:** WOMAC pain progression.

Progression definition	Cycles 1–2 (Weighted N = 108.6)	Cycles 2–3 (Weighted N = 108.6)	Cycles 1–3 (Weighted N = 121.2)
Delta WOMAC, mean (SD)	-4.3 (16.0)	4.5 (17.0)	0.7 (18.5)
Delta WOMAC 10+, n (%)	15.3 (14.1)	27.2 (25.0)	27.0 (22.3)
Delta WOMAC 20+, n (%)	7.5 (6.9)	19.3 (17.7)	17.8 (14.7)

Table 3 lists the coefficients and 95% confidence intervals (CIs) from the cross-sectional linear regression models, adjusted for age, sex and BMI. Significant predictors of WOMAC pain cross-sectionally included osteophytes (the regression coefficient representing the effect per unit region-average osteophyte grade across regions was 7.17 WOMAC pain units; 95% CI = 3.19, 11.15), and subchondral sclerosis (11.03; 0.68, 21.39). Choice of the exchangeable working correlation matrix in these models was validated according to lowest QIC (compared to an unstructured matrix). In sensitivity analyses further adjusted for NSAIDs, acetaminophen and PT use, statistical significance in these models did not differ.

**Table 3:** Cross-sectional linear models of WOMAC pain.

MRI feature	Cross-sectional linear regression coefficient (95% CI)
Osteophytes (sum/8)	7.17 (3.19, 11.15)
Cartilage (sum/6)	2.80 (-0.70, 6.30)
Subchondral sclerosis (sum/6)	11.03 (0.68, 21.39)
Subchondral cysts (sum/6)	5.13 (-7.43, 17.68)
BML (sum/6)	2.12 (-3.45, 7.69)
Meniscus (sum/6)	1.47 (-1.33, 4.27)
Effusion/synovitis ≥2	-2.40 (-6.25, 1.45)

Models are adjusted for age, sex and BMI

Table 4 lists the odds ratios (ORs) and 95% CIs for the longitudinal binary logistic models, adjusted for interval-baseline age, sex and BMI, plus cycle-to-cycle years of follow-up. Significant predictors of a 10+ increase in WOMAC pain included osteophytes (the odds ratio, representing the effect per unit region-average osteophyte score, was 3.20; 95% CI = 1.36, 7.55), subchondral sclerosis (5.69; 1.06, 30.44), meniscus (1.68; 1.08, 2.61), and presence of effusion score ≥2 (2.25; 1.07, 4.71). Significant predictors of a 20+ increase in WOMAC pain included osteophytes (OR = 3.79; 95% CI = 1.41, 10.20) and cartilage (2.42; 1.24, 4.74). All models passed the Hosmer and Lemeshow GOF test at alpha = .05. In sensitivity analyses further adjusted for NSAIDs, acetaminophen and PT use, the direction of effects and statistical significance in these models did not differ.

**Table 4:** Longitudinal logistic models of delta WOMAC pain.

MRI feature	Odds ratio for delta 10+ (95% CI)	Odds ratio for delta 20+ (95% CI)
Osteophytes (sum/8)^a^^b^	3.20 (1.36, 7.55)	3.79 (1.41, 10.20)
Cartilage (sum/6)^b^	1.66 (0.95, 2.89)	2.42 (1.24, 4.74)
Subchondral sclerosis (sum/6)^a^	5.69 (1.06, 30.44)	1.63 (0.18, 14.60)
Subchondral cysts (sum/6)	4.86 (0.72, 32.65)	5.99 (0.51, 71.13)
BML (sum/6)	1.38 (0.55, 3.49)	0.87 (0.23, 3.27)
Meniscus (sum/6)^a^	1.68 (1.08, 2.61)	1.12 (0.71, 1.75)
Effusion/synovitis ≥2^a^	2.25 (1.07, 4.71)	1.24 (0.47, 3.26)

Models are adjusted for interval-baseline age, sex, BMI, and cycle-to-cycle follow-up time

^a^ Feature predicts 10+ WOMAC pain increase

^b^ Feature predicts 20+ WOMAC pain increase

Our further exploration of full thickness cartilage lesions on individual surfaces revealed that a full thickness lesion was only borderline (p = 0.054) associated with WOMAC pain cross-sectionally on one of the six surfaces (medial-femoral), when adjusted for age, sex and BMI. This is consistent with the continuous model shown in Table 3. Full thickness cartilage lesion on the same surface (medial-femoral) was a significant predictor of both 10+ and 20+ changes in pain. Full thickness cartilage lesions on the other five surfaces were not statistically significantly associated with pain in either cross-sectional or longitudinal adjusted models.

The results of the sensitivity analyses exploring possible alternatives to our aggregate osteophyte score (one eliminating medial/lateral patellar surfaces, the other eliminating superior/inferior patellar surfaces) were consistent with the primary analysis, both in direction of effects and statistical significance of the osteophyte variables.

Finally, there were no statistically significant differences between the followed up subsample of 122/255 vs. the subsample that was not followed up (133/255), on baseline values of the MRI features of interest, age sex and BMI (data not shown).

## Discussion

In models adjusted for age, sex, BMI and, where applicable, years of follow-up, we investigated the association between knee pain severity (cross-sectional models) and progression (longitudinal models) versus the MRI features osteophytes, cartilage, subchondral sclerosis, subchondral cysts, bone marrow lesions, meniscus and effusion/synovitis, in a population-based cohort with knee pain.

Cross-sectionally, the MRI features we found to be associated with pain severity included osteophytes and subchondral sclerosis. Several previous cross-sectional studies also found associations between pain severity and one or both of these features.[11, 15, 16, 18, 20, 22] However, these studies did not directly analyze the WOMAC pain scale, and therefore the specific estimates could not be compared to ours. These studies also analyzed dichotomous presence of pain rather than severity, thus their results do not translate into analysis of pain severity. In addition, as discussed in the introduction, there is some disagreement among previous studies both in how pain variables are defined, as well as models and conclusions.

While many patients experienced pain progression, other did not, and some pain levels actually improved over time. Longitudinally, the MRI features we found to be associated with pain progression were dependent on the threshold for delta WOMAC pain used to define "progression", with cut points of either 10+ (based on the minimum perceptible clinical improvement or MPCI) or 20+ (based on the minimum clinically important difference or MCID). Statistically significant predictors in at least one of the pain progression models included osteophytes, cartilage defects, subchondral sclerosis, meniscal damage and effusion. Two previous longitudinal studies were identified finding associations between pain and one or more of these features.[8, 9] Neither study analyzed progression on the WOMAC pain scale, although Javaid et al [9] reported odds ratios for pain incidence similar in magnitude to our findings. They reported an OR of 4.7 (1.3, 18) for the effect of severe osteophyte vs. no osteophyte on incident knee pain. This is comparable to our OR of 3.79 (1.41, 10.20) for the effect per unit region-average osteophyte score on MCID-based WOMAC pain progression, the interpretation being that more severe osteophytes relate to a greater frequency of large-scale pain progression.

It is noteworthy in our study that among the MRI predictors of pain progression, only osteophytes were statistically significant in both the MPCI- and MCID-based models. Subchondral sclerosis, meniscal damage and effusion/synovitis were only statistically significant in the MPCI-based models, which may suggest that these processes alone (without additional factors present), while detectable to the patient, are not sufficient to induce large increases in pain. Conversely, cartilage is only statistically significant in the MCID-based model, and while borderline significant (p<0.10) in the MPCI-based model, there it presents less than half the added risk seen in the MCID-based model. This may be explained by the fact that cartilage is aneural and avascular, and hence minor cartilage defects alone are insufficient to directly induce pain, until they become severe/full-thickness, at which point the underlying bone is exposed to an extent sufficient to effect large increases in pain (i.e., an "on-off" switch for more severe pain). Consistent with this interpretation are the findings of Sowers et al [11] (a cross-sectional analysis of 724 knee MRIs) in which a significant cartilage-pain association was only evident with full-thickness defects. (In our sensitivity analyses exploring full thickness cartilage lesions on individual surfaces, we found one surface [medial-femoral] to be statistically significantly associated with both cross-sectional and longitudinal pain outcomes.) In a narrative review, Hunter et al [23] also cited exposed bone as the primary mechanism in the cartilage-pain association, describing as secondary mechanisms associations between cartilage defects and other structural changes (e.g., BML or effusion). Finally, also consistent with this interpretation is our finding that osteophytes are statistically significant in both MPCI- and MCID-based pain progression models; the continuum of minor to severe osteophytes could allow for a graduated boney involvement in knee pain. Collectively, these findings might suggest that the single most consistent (direct) cause of pain in knee OA, among those we studied, involves bone.

While some previous studies reported unadjusted (crude) results, the majority of studies (as we did) adjusted for age, sex and BMI.[9, 12–17, 20, 21, 25, 30, 32] A small number adjusted also for drug treatment,[12] depression,[17] occupation,[20] or history of joint injury.[20] To investigate the potential impact of certain potential additional adjustments on our findings, in sensitivity analyses we further adjusted all models for current regular NSAID or acetaminophen use, and current or recent PT use. In these sensitivity analyses, the direction of effects and statistical significance of the MRI features did not differ.

The limitations of our study deserve comment. While our study is population-based, it should be noted that the target population is not the overall population, but those with baseline knee pain, aged 40–79 at baseline, who were successfully followed up over an average of 7.5 years. As such, we cannot be sure that the results of this study are applicable to a more general population that includes people without baseline knee pain, or those outside the target age range of 40+. Another important limitation of this study is loss to follow-up; only 122 of the original 255 baseline subjects were successfully followed through to the third cycle. To explore the effect this might have had, we analyzed potential differences between those followed vs. not followed on age, sex, BMI, and the six MRI scores (at baseline) analyzed in this study, and found no statistically significant difference. Related to this is the sample size itself (N = 122). There are larger datasets on which similar analyses might be undertaken, for example the Osteoarthritis Initiative, and it would be prudent in future research to perform comparative analyses on these data. Another limitation of this study is that MRIs, although scored by a highly experienced, board-certified musculoskeletal radiologist (AG), were nevertheless not scored with recent semi-quantitative methods like the BLOKS/WORMS or MOAKS. This is because such methods were not yet available at the time of our baseline readings. However, the scoring methods we employed were based in part on earlier studies, e.g., Disler et al.[33] Despite these limitations, this is one of the few studies to report results from both cross-sectional and longitudinal models together from the analysis of a common data set, and the first such bi-modal study to report effects on pain severity cross-sectionally rather than simple presence of knee pain. This study therefore offers a more complete picture of the implications of MRI features on knee pain as a whole—both cross-sectional severity (correlation) as well as longitudinal progression (prediction).

In summary, of the MRI features we investigated, only osteophytes were consistently associated with pain cross-sectionally (severity models) as well as longitudinally (MPCI- and MCID-based pain progression models). This suggests an important role of bone in early knee osteoarthritis.
